# P-1368. Tuberculous Meningitis: A 30-Year Retrospective Analysis of Clinical, Epidemiological, and Radiological Insights

**DOI:** 10.1093/ofid/ofaf695.1555

**Published:** 2026-01-11

**Authors:** Amal Chakroun, Fatma Hammami, khaoula Rekik, Makram Koubaa, fatma smaoui, chakib marrakchi, Mounir Ben Jemaa

**Affiliations:** Infectious Diseases Department, sfax, Sfax, Tunisia; Infectious Diseases Department, Hedi Chaker University Hospital, University of Sfax, Tunisia, Sfax, Sfax, Tunisia; Infectious Diseases Department, sfax, Sfax, Tunisia; Infectious Diseases Department, Hedi Chaker University Hospital, University of Sfax, Tunisia, Sfax, Sfax, Tunisia; Infectious Diseases Department, sfax, Sfax, Tunisia; Infectious Diseases Department, sfax, Sfax, Tunisia; Infectious Diseases Department, Hedi Chaker University Hospital, University of Sfax, Tunisia, Sfax, Sfax, Tunisia

## Abstract

**Background:**

Tuberculous meningitis (TBM) is a life-threatening condition characterized by significant clinico-radiological heterogeneity. The challenges in achieving a definitive diagnosis, along with ongoing debates regarding treatment strategies, make it a complex condition for clinicians to manage. We aimed to describe the epidemiological and clinical features of TBM, assess the contribution of radiological techniques in diagnosis, and evaluate the management approaches and clinical outcomes of this disease.

**Methods:**

We conducted a retrospective, descriptive analysis of the medical records of patients hospitalized for TBM in the Infectious Diseases Department at Hedi Chaker University Hospital, Sfax, over a 30-year period (1994–2024)

**Results:**

A total of 105 cases were included in the study. The median age of patients was 35 years (IQR = [25-50.5]), with a male-to-female ratio (M/F) of 0.9. The majority of patients (58.1%, n = 61) came from rural areas. Fever was the most common presenting symptom, observed in 92 patients (87.6%). Neurological examination revealed neck stiffness in 50 patients (47.6%). Cerebrospinal fluid (CSF) analysis demonstrated hypoglycorrhachia in 78 cases (74.3%) and hyperproteinorhachia in 96 cases (91.4%). A definitive diagnosis was established through positive BK PCR testing on CSF in 23 patients (22%). Brain CT scans, performed in 67 patients (63.8%), were normal in 46 patients (68.7%), with tuberculomas identified in 13.3% of cases. Cerebral MRI, which is more sensitive, was performed in 40 patients (38.1%) and revealed abnormalities in all but 2 patients (1.9%), with arachnoiditis seen in 20% of cases. The treatment regimen consisted of quadruple therapy in 101 patients (97.2%), with a median treatment duration of 360 days (IQR = [17-540] days). The overall mortality rate was 10.5%

**Conclusion:**

Early diagnosis of tuberculous meningitis is significantly enhanced by the use of PCR and cerebral MRI. However, the diagnosis still primarily relies on a combination of epidemiological, clinical, biological, and radiological findings. The duration of anti-tuberculosis therapy remains extended in most cases, highlighting the importance of long-term management strategies.

**Disclosures:**

All Authors: No reported disclosures

